# MRI-based delta-radiomic features for prediction of local control in liver lesions treated with stereotactic body radiation therapy

**DOI:** 10.1038/s41598-022-22826-5

**Published:** 2022-11-03

**Authors:** Will H. Jin, Garrett N. Simpson, Nesrin Dogan, Benjamin Spieler, Lorraine Portelance, Fei Yang, John C. Ford

**Affiliations:** 1 Department of Radiation Oncology, Jackson Memorial Hospital, Miami, USA; 2 Department of Radiation Oncology, Sylvester Comprehensive Cancer Center, University of Miami, Miami, USA

**Keywords:** Outcomes research, Cancer imaging, Predictive markers

## Abstract

Real-time magnetic resonance image guided stereotactic ablative radiotherapy (MRgSBRT) is used to treat abdominal tumors. Longitudinal data is generated from daily setup images. Our study aimed to identify delta radiomic texture features extracted from these images to predict for local control in patients with liver tumors treated with MRgSBRT. Retrospective analysis of an IRB-approved database identified patients treated with MRgSBRT for primary liver and secondary metastasis histologies. Daily low field strength (0.35 T) images were retrieved, and the gross tumor volume was identified on each image. Next, images’ gray levels were equalized, and 39 s-order texture features were extracted. Delta-radiomics were calculated as the difference between feature values on the initial scan and after delivered biological effective doses (BED, α/β = 10) of 20 Gy and 40 Gy. Then, features were ranked by the Gini Index during training of a random forest model. Finally, the area under the receiver operating characteristic curve (AUC) was estimated using a bootstrapped logistic regression with the top two features. We identified 22 patients for analysis. The median dose delivered was 50 Gy in 5 fractions. The top two features identified after delivery of BED 20 Gy were gray level co-occurrence matrix features energy and gray level size zone matrix based large zone emphasis. The model generated an AUC = 0.9011 (0.752–1.0) during bootstrapped logistic regression. The same two features were selected after delivery of a BED 40 Gy, with an AUC = 0.716 (0.600–0.786). Delta-radiomic features after a single fraction of SBRT predicted local control in this exploratory cohort. If confirmed in larger studies, these features may identify patients with radioresistant disease and provide an opportunity for physicians to alter management much sooner than standard restaging after 3 months. Expansion of the patient database is warranted for further analysis of delta-radiomic features.

## Introduction

Stereotactic body radiation therapy (SBRT) is an established treatment option for both primary hepatocellular carcinoma (HCC), intrahepatic cholangiocarcinomas (ICC) and metastatic lesions within the liver^1–4^. In primary HCC, Magnetic resonance guided (MRg) linear accelerators (linacs) are being explored as a complementary tool to support the patient prior to definitive hepatectomy or transplant. Without definitive resection, 5-year overall survival is estimated at less than 10%^5^. Improving progression free survival acts to bridge patients until definitive treatment, another line of chemotherapy or in the palliative setting. In metastatic liver disease, it offers a safe and convenient way to obtain durable local control for metastasis-directed therapies (MDT)^3,6,7^. MRgSBRT provide an ideal platform for treating abdominal lesions due to its distinct advantages over standard-of-care cone beam computerized tomography (CBCT) image guidance. First, the superior soft tissue visualization of MR images eliminates the need for fiducial marker placement required for localizing oncologic targets in CBCT imaging. Second, MRg linacs provide real-time tracking during treatment with automatic beam control. Motion management remains a challenge in abdominal tumors due to the proximity to the diaphragm, and this feature automatically turns off the beam when the target is out of range. Third, there is no unnecessary ionizing radiation delivered when daily imaging is done on the MR system. Finally, a fourth, yet unquantified benefit may come from analyzing the imaging data generated from daily image-guidance.

Despite low field strengths, the images generated from daily MR set up images have adequate soft tissue contrast to visualize targets and organs at risk (OAR). During the last decade, the field of oncology moved towards personalized medicine^8^, and radiomics presented itself as a pathway for imaging to potentially guide personalized management decisions. Radiomics texture analysis quantifies the spatial relationships of individual voxel characteristics within a region of interest^9^. These statistical descriptors served as imaging biomarkers used in models for predicting toxicities, risk stratification, and oncologic outcomes^10–17^.

Delta-radiomics expands upon texture analysis by considering changes in features induced by treatments. Delta-radiomics is gaining traction and initial studies seem promising. Fave et al. initially postulated that predictive information can be extracted from CBCT set up images in lung cancer patients^18^, while Boldrini et al. applied this concept to low field strength MR setup images and correlated delta-radiomics texture values with response to neoadjuvant therapy in rectal cancer patients^19^. Neoadjuvant therapy consists of combination chemoradiation, with the goal to shrink tumors as much as possible for a potential surgery. In the setting of SBRT, we suspect that changes in texture features proxy different responses to radiation. By extracting features from multiple timepoints, phenotypic changes in the tumor microenvironment during treatment can be captured and used for predictive modelling^15,20^.

We hypothesize two theoretical strengths with delta-radiomics. First, data required for analysis is readily available since it comes directly from MRgSBRT. Secondly, one can potentially predict for treatment response much sooner than with standard of care imaging protocols. Treatment response is normally evaluated at least three months after treatment via Response Evaluation Criteria in Solid Tumors (RECIST) or modified RECIST (mRECIST)^21^. These grading systems primarily rely on size of index lesions or intensity of metabolic imaging. This much time is needed to allow for adequate resolution of non-pathologic inflammatory changes incurred from ablative radiation. If treatment response were available immediately at the end of treatment, clinicians potentially gain valuable time to adjust management strategies like coordinating other MDT for metastatic liver disease or systematic therapies for primary HCC/ICC. The purpose of this work is to explore delta radiomics textures as predictive markers for liver lesion treatment response using MRgSBRT. The delta radiomics texture features were calculated after delivery of dose based on the total biological effective dose (BED) delivered to each patient based on each patient’s prescription. We hypothesize that underlying biological changes induced by delivery of radiation will be captured and quantified by the delta radiomics texture features and may be predictive of local control.

## Methods

### Patient selection

Retrospective analysis of an IRB-approved database of patients treated on our MRg system was done to identify patients with liver lesions treated with MRgSBRT. Patients were included if they had a biopsy-proven primary HCC, known history of a primary malignancy with biopsy-proven metastasis, or consensus to treat after review in a multidisciplinary tumor board. Patients were excluded from analysis if they had synchronous primary malignancies, did not complete MRgSBRT, or did not have post-treatment imaging for restaging. Data abstracted included demographics, performance status, history of cirrhosis, history of hepatitis C infection, prior radiofrequency ablations, prior radiation treatments, prior chemotherapy regimens, prior trans-arterial chemoembolization procedures, pre-treatment complete metabolic panels, pre-treatment complete blood counts, tumor size, tumor location in the liver, histology, radiation dose delivered, and radiation fractions delivered.

### Image acquisition and target delineation

**Figure 1:**
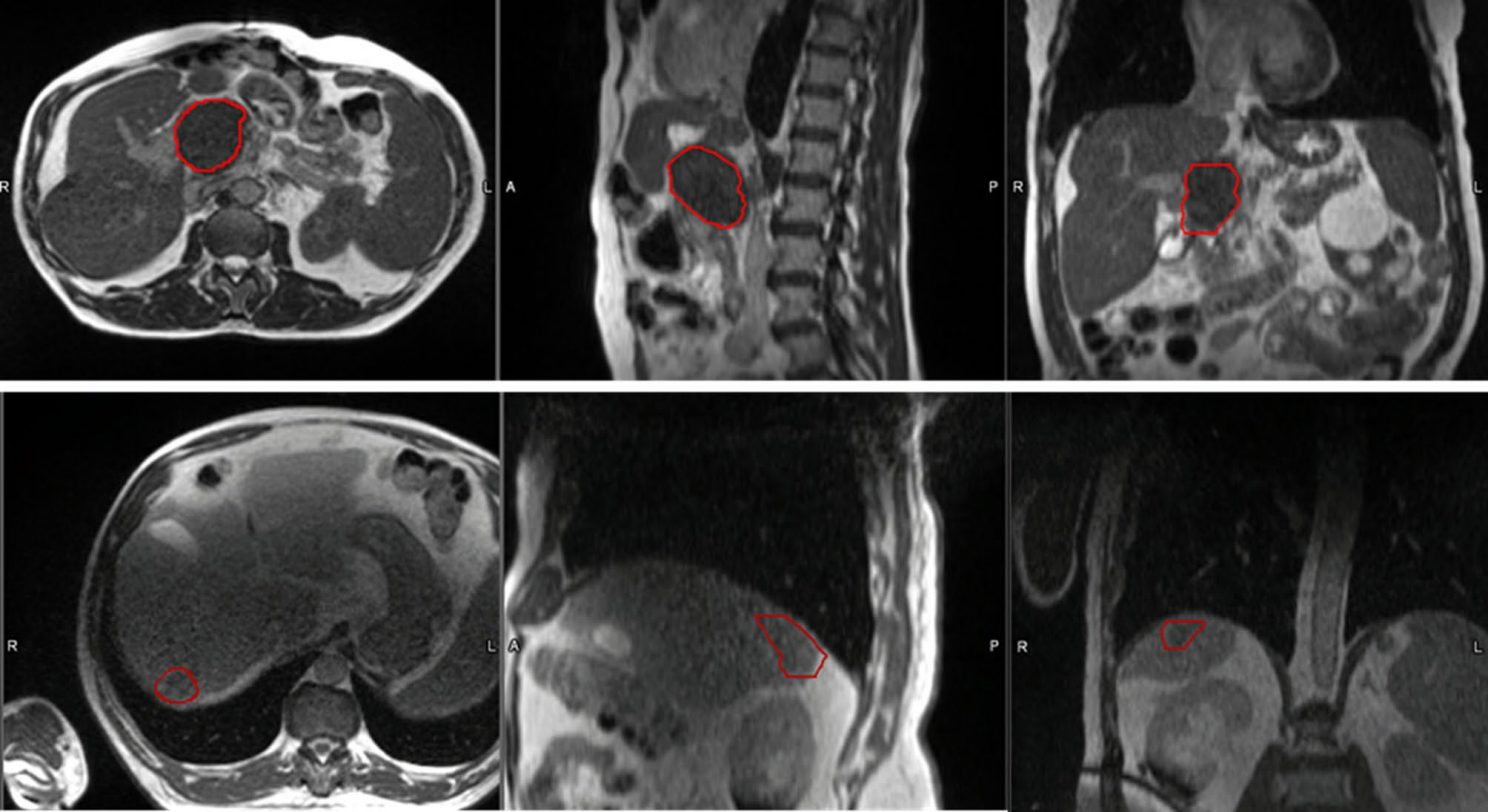
Target volume delineation example. The image displays two patient GTVs contoured on the fraction 1 set up scan. Each row is a different patient with the GTV contoured in red. From left to right, axial, sagittal, and coronal views are displayed, respectively.

Low field strength setup MR images acquired on a 0.35 T split bore unit using a balanced steady-state free procession pulse sequence were retrieved from the MRg Linac system (MRIdian, ViewRay Inc., Mountain View, CA)^22^. The image acquisition protocol was similar for all images acquired during treatment; breath-hold images were acquired in 25 s with voxel dimensions of 1.5 × 1.5 × 3.0 mm^3^. Two torso coils with six-phased array elements were placed hemi-circumferentially around the patient. Same day computed tomography (CT) simulation was performed with MR simulation. Images were fused and co-registered with diagnostic CT (n = 11), PET/CT (n = 7) or MR (n = 4). Treatment proceeded only if a GTV was visualized through a combination of image registrations (Fig. 1). MRI safety screening were administered to all eligible patients. Images were acquired prior to each daily fraction for patient set up prior to treatment delivery. For fractions adapted online, the GTV was rigidly transferred to the daily set up image after fusion by physics. OARs are deformably transferred and contoured by the physicist. The GTV and OARs are reviewed by the attending radiation oncologist who may manually adjust volumes based on his/her clinical discretion. A 3 mm margin along with any expansion rules is then added to obtain the planning treatment volume. For the analysis the Gross Tumor Volume (GTV) was delineated by a radiation oncologist resident and reviewed by a board-certified radiation oncologist experienced in MRgSBRT and low field MR setup images. Texture features were extracted from the GTVs only. Treatment response within 12 months of MRgSBRT was assessed using RECIST and mRECIST criteria, depending on initial histology. Poor treatment response included target lesions with progressive disease only; otherwise, complete responses, partial responses, and stable disease were considered responders.

### Image analysis, radiomic feature selection, and model construction

Image DICOM files along with the associated GTV structure sets were exported to a local drive and radiomics texture features were extracted using the Texture Analysis Toolbox in MATLAB 2020b (The MathWorks, Natick MA, USA). Binary masks of the GTVs were generated to extract three-dimensional bitmaps from the RTStruct DICOM objects. The dynamic range of intensity values was constrained via “± 3σ” Collewet normalization method^23^. The intensity values were then quantized to 64 levels using histogram equalization^24^. A total of 39 s-order features were extracted from the GTVs of the daily MR setup images. Feature classes utilized were gray level co-occurrence matrix (GLCM), gray level size zone matrix (GLSZM), gray level run length matrix (GLRLM), and neighborhood gray tone difference matrix (NGTDM)^25–29^. The GLCM code/aggregation code for calculation of the GLCM was LFYI/IAZD with the feature name 8ZQL when using the Image Biomarker Standardization Initiative (IBSI) nomenclature^30^. The IBSI code/aggregation code for the GLSZM is 9SAK/KOBO and the feature name and formula is 48P8. Prior to calculation of delta-radiomic texture features, radiomics texture features extracted from fraction 1 set up images were compared using Student’s t-test to look for significant differences between patients with local failure and those without local failure. The Bonferroni correction was applied to account for multiple tests with significance was set to be *p* values ≤ 0.05. Delta-radiomic texture features were calculated as the change in radiomic features between the initial setup image (before treatment) and after BEDs of 20 Gy and 40 Gy were delivered, producing BED_20_ and BED_40_ feature libraries. These quanta of radiation were selected to account for dissimilar radiation doses and fractionation schedules. For example, a delta-radiomics texture feature value calculated from a hypothetical patient with a BED/Fx = 20 Gy would be calculated for the BED_20_ with the texture features calculated from the image acquired prior to fraction 1 and the setup image from fraction 2 (delivery of 1 treatment).

### Model construction and feature selection

A two-step method was used where the first step was designed to rank features in order of predictive potential, while the second step was used to evaluate the top features. In step one, features were ranked using the Gini Index, calculated during the training phase of a random forest (RF) model. The Gini Index is a measure of how important each predictive variable is for accurate prediction^31^. A RF model was built using delta-radiomics as predictors with 500 decision trees considering six features (‘mtry’ parameter) with replacement one at a time. The ‘mtry’ parameter was selected as approximately the square root of the number of delta-radiomics texture features under consideration, thirty-nine total for each timepoint. The RF prediction model selects subsets of the available data for predictors, constructs a simple decision tree, then evaluates the accuracy of predictors included and excluded from each decision tree. This is performed for each tree in the forest. The importance of the variables in the model for prediction is calculated and recorded as the Gini Index. Finally, the two features with the highest Gini Index were selected for further investigation.

Those two features were used to estimate the internal validation area under the receiver operating characteristic curve (AUC) using a bootstrapped logistic regression model. The model was bootstrapped 1,000 times randomly sampling 2/3 of the patient data at each iteration. The internal validation AUC was obtained by feeding in the remaining 1/3 of the patients and predicting response. The AUC was recorded for each iteration. This process was performed for both BED_20_ and BED_40_ features that were selected by the Gini Index. The mean AUC, 2.5 percentile, and 97.5 percentile values were calculated using the 1,000 iterations. Two additional random forest models were constructed, a strictly clinical model, and a combined model (delta-radiomic BED_20_ features with clinical data), to compare the importance of clinical variables and BED_20_ delta-radiomic texture features using the Gini Index. The clinical model included BED/fraction, maximum tumor dimension, sex, ethnicity, initial Karnofsky performance status score, and existence of prior cirrhosis as variables for predicting treatment response. The AUC estimates produced by the random forest model during training was recorded for evaluation. The process was repeated for the combined model with the top two BED_20_ delta-radiomics features included to assess the importance of the factors relevant to available clinical information. Both the clinical model and combined models were constructed with 500 trees. The mtry parameter was set to include all data (mtry = 6 for the clinical model and mtry = 8 for the combined model). Reproducibility of features were confirmed using Pfaeler’s criteria^32^.

### Ethics declaration

All methods were carried out in accordance with relevant guidelines and regulations.

The study was approved by The University of Miami’s institutional IRB committee under IRB#20,160,817. Informed consent was waived due to the retrospective nature of the study by The University of Miami’s institutional IRB.

## Results

### Patient and treatment characteristics

A total of 22 patients were identified in the database. The median age of patients was 73 years old, ranging from 49 to 94 years old (Table 1). Treatment regimens consisted of 3–5 fractions delivering physical doses of 30 Gy to 60 Gy. The median BED, calculated with an α/β = 10, was 100 Gy, ranging from 48 to 180 Gy^21^. The median BED/Fx was 20 Gy with a range from 9.6 to 60 Gy/Fx. Median follow-up for the entire cohort was 13.98 months. 2-year actuarial local control and survival were 14.6 months and 18.65 months, respectively. See Table 1 for relevant clinical information.

**Table 1:** Patient variables included for delta-radiomics texture analysis.

Patient number	Poor treatment response	Age (years)	Rx (Gy)	Fractions	Total BED	BED/Fx	Tumor size in largest dimension (cm)	Diagnosis
1	Yes	66	45	5	85.5	17.1	4.3	HCC
2	No	62	50	5	100	20	2	LM
3	No	70	50	5	100	20	7.8	HCC
4	Yes	66	45	5	85.5	17.1	1.3	LM
5	Yes	56	50	5	100	20	3.1	LM
6	Yes	74	50	5	100	20	2.1	LM
7	No	92	50	5	100	20	7.2	HCC
8	No	81	40	4	80	20	1.8	LM
9	No	75	50	5	100	20	3.9	HCC
10	No	79	50	5	100	20	4.3	ICC
11	No	70	50	5	100	20	2.7	HCC
12	No	90	50	5	100	20	6.4	HCC
13	Yes	49	30	5	48	9.6	8	LM
14	No	84	50	5	100	20	7.3	LM
15	No	54	50	5	100	20	2.5	HCC
16	Yes	72	60	5	132	26.4	2.4	LM
17	No	87	54	3	151.2	50.4	1.8	LM
18	No	59	30	3	60	20	2.7	HCC
19	Yes	50	50	5	100	20	4.8	LM
20	No	72	60	5	132	26.4	3.3	LM
21	No	71	50	5	100	20	1.7	LM
22	No	91	50	5	100	20	4.7	ICC

BED, biologically effective dose (calculated using α/β = 10); HCC, hepatocellular carcinoma; ICC, intrahepatic cholangiocarcinoma; LM, secondary liver metastases.

### Delta radiomics feature selection

**Figure 2:**
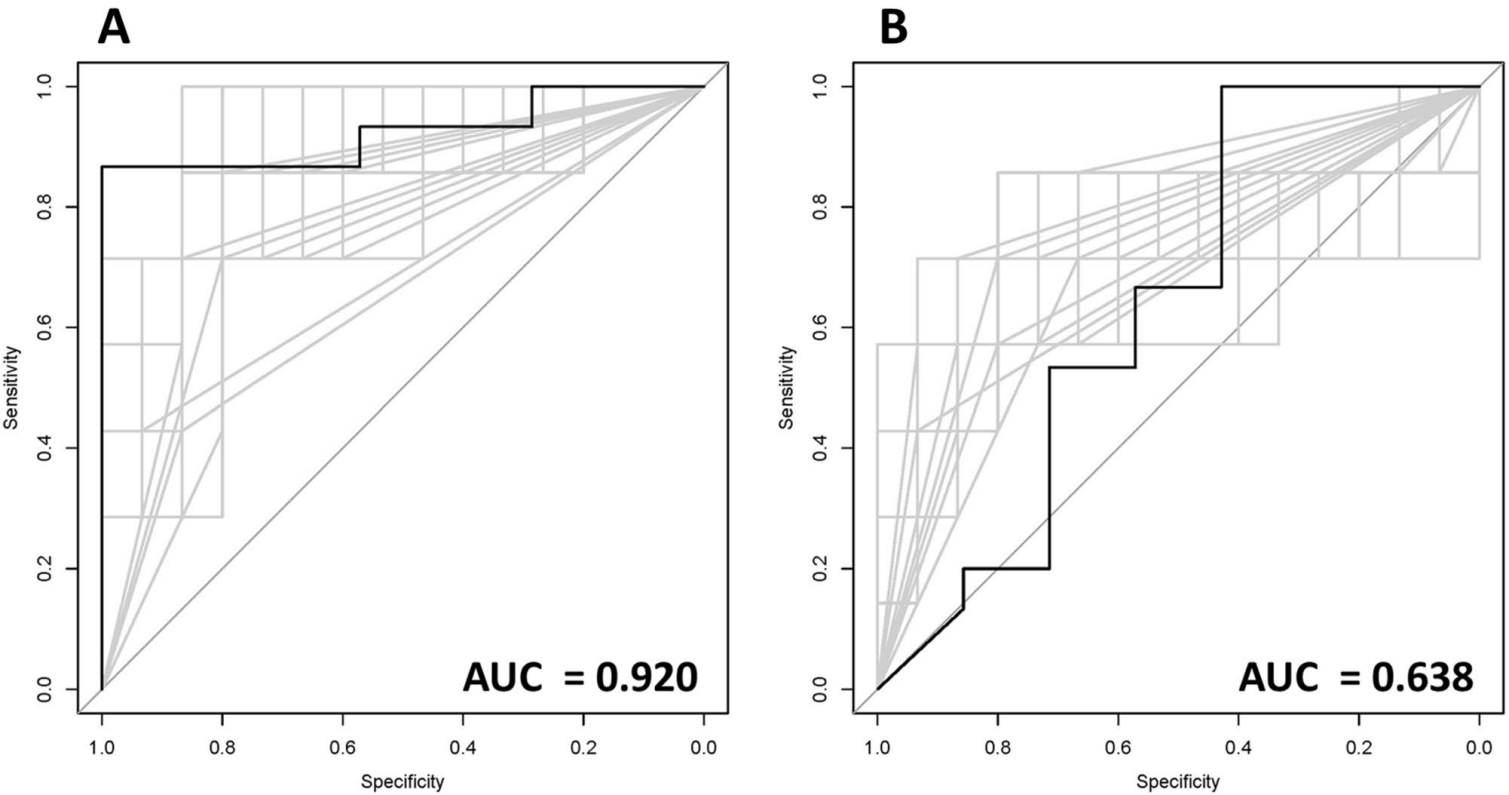
The bolded receive operator characteristic curve in each graph represents the random forest's estimate obtained during training for (**A**) BED_20_ and (**B**) BED_40_. The AUC for the BED_20_ delta-radiomics features = 0.920 and the AUC for the BED_40_ was = 0.638.

The Student’s t-test of each texture feature extracted from fraction 1 set up images resulted in no significant differences with the Bonferroni correction, all *p* values > 0.05. The two features selected for the BED_20_ library were gray level co-occurrence matrix (GLCM) energy and gray level size zone matrix (GLSZM) based large zone emphasis. During model training, the estimated AUC for the BED_20_ delta-radiomics features was 0.920 (Fig. 2). Based on the selected delta-radiomics BED_20_ features, the bootstrapped logistic regression achieved a mean AUC = 0.901 with the 2.5 percentile–97.5 percentile range = 0.752–1.0 (Table 2). The BED_40_ delta-radiomic texture feature model selected the same two features but calculated a higher Gini Index for gray level size zone matrix (GLSZM) large zone emphasis than for energy (GLCM). During model training, the estimated AUC for the BED_40_ delta-radiomics features was 0.638 (Fig. 2). The bootstrapped logistic regression resulted in an AUC = 0.716 with the 2.5 to 97.5 percentile range = 0.600 to 0.786 using the selected BED_40_ delta-radiomic texture features (Table 2).

**Table 2:** Performance of delta-radiomics texture features ranked by the Gini Index using bootstrapped logistic regression analysis for each library.

Delta-radiomics library	Highest Gini Index Feature	Second Highest Gini Index Feature	Mean AUC	2.5 Percentile Value	97.5 Percentile Value
BED_20_	Energy	Large Zone Emphasis	0.901	0.752	1.0
BED_40_	Large Zone Emphasis	Energy	0.716	0.600	0.786

AUC, area under the curve; BED, biologically effective dose (calculated using α/β = 10).

**Figure 3:**
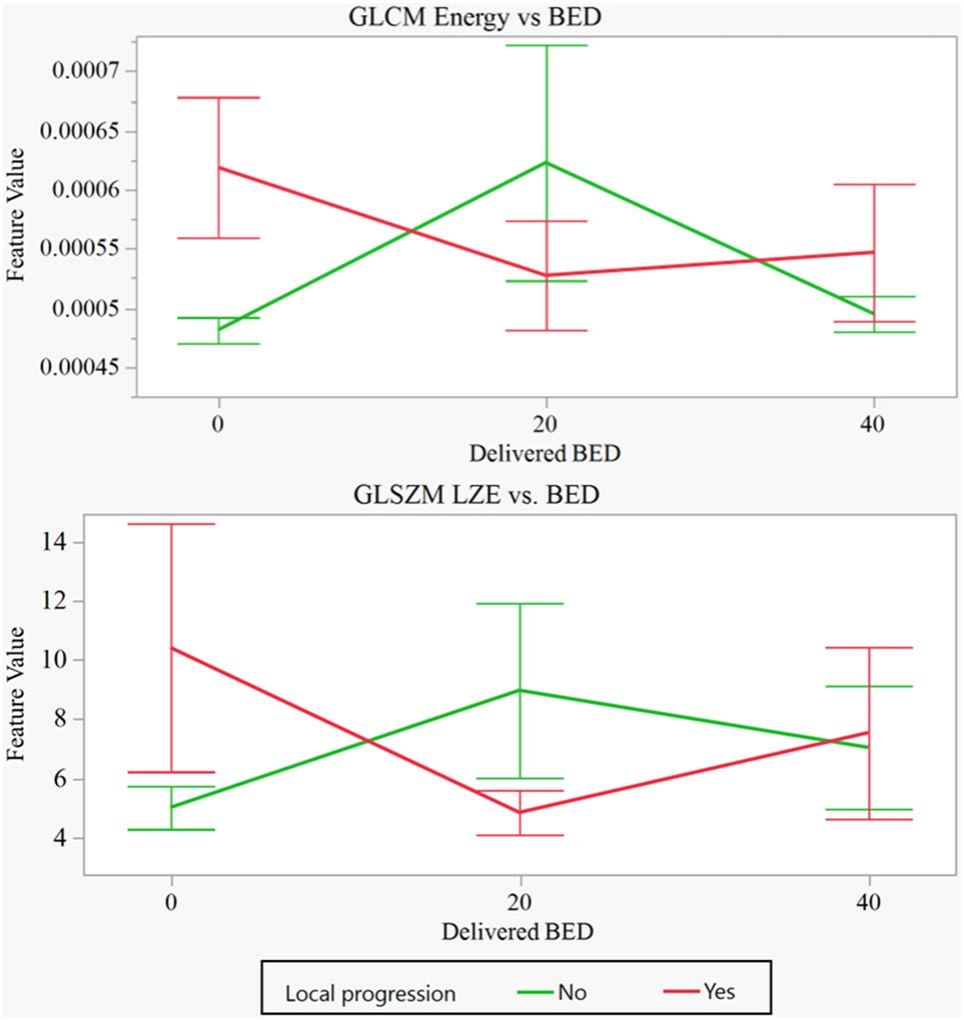
Mean feature values for patients prior to delivery (BED = 0 Gy), after delivery of BED 20 Gy, and after BED 40 Gy. Both radiomics texture features for the patients without local progression behaved similarly with increased values from BED 0 Gy to BED 20 Gy and decreasing from BED 20 Gy to the BED 40 Gy point (in green). The patients with local progression displayed the opposite, feature values initially decreased from BED 0 to BED 20 then increased from BED 20 Gy to BED 40 Gy. With no multiple test statistical comparisons GLCM energy extracted from the first set up image had a *p* value ≤ 0.05 at BED 0 Gy point. However, after applying the Bonferroni correction, it was not statistically significant (*p* value > 0.05). BED = biologically effective dose (calculated using α/β = 10), GLCM: gray level co-occurrence matrix, GLSZM: gray level size zone matrix, LZE: large zone emphasis. The vertical bars represent the standard error of each group.

The behavior of the features for patients with local control and those with local failure can be seen in Fig. 3. Both radiomics texture features for the patients without local progression behaved similarly with increased values from BED 0 Gy to BED 20 Gy and decreasing from BED 20 Gy to the BED 40 Gy point. The patients with local progression displayed the opposite, feature values initially decreased from BED 0 to BED 20 then increased from BED 20 Gy to BED 40 Gy.

### Combined clinico-radiomics modeling

For clinical features, cohort characteristics in Table 1 utilized for analysis were age, total BED, BED/fraction, and tumor size in largest dimension. The random forest model was run using the same method to select important features as mentioned above and selected maximum tumor dimension as most important, followed by BED/fraction. The model estimated the AUC = 0.724 with 95% confidence interval = 0.446–1.0. The combined model ranked GLCM energy as most important for predictive accuracy, followed by GLSZM large zone emphasis, then tumor size, and BED/fraction. Patient age and the total BED of the treatment course were ranked lowest and not important for accurate prediction. Inclusion of the BED20 delta-radiomics texture features to the clinical model improved the performance estimation with an AUC = 0.895 with the 95% confidence interval = 0.762–1.0.

## Discussion

To our knowledge, this is the first study evaluating delta-radiomic features of daily adaptive MR imaging in liver lesions treated with SBRT. Comparison of fraction 1 radiomics texture features resulted in no significant differences between the two groups of patients after applying the Bonferroni correction, but GLCM energy and GLSZM large zone emphasis, were identified by the model as possibly predictive of local control. Features identified earlier in treatment (BED_20_) performed better than those using features later (BED_40_) in treatment. Interestingly, the combined clinico-radiomics model did not perform better than the BED_20_ model alone. If validated in prospective studies, delta-radiomics could drastically reduce the time clinicians need to make critical changes in management decisions. In primary HCC, early detection of poor responsivity to radiation can give clinicians enough time to prepare additional bridging therapies. In metastatic cases, delta-radiomics may provide an assurance to switch lines of systemic therapy. The earlier we can detect an actionable signal among the noise, the more benefit we provide to our patients.

Other studies reported different features and varying optimal times for evaluating such features. Gemelli University produced two series of adaptive delta radiomics studies on a 0.35 T on-board MR-linac, in a collaboration with University of Wisconsin. In their LARC cohort, they were able to predict pCR using two radiomic features, L_least_ and glnu^33,34^. In their locally advanced pancreatic cancer cohort, texture features like GLCM, GLRLM, and GLDZM predicted for crude 1-year local control with an AUC of 0.79^35^. These texture features were predictive of local control after BED_40Gy_ was delivered, equivalent to a complete course of 5 fractions. Most prior studies evaluated the utility of delta features between pre- and post-treatment images. In MRgSBRT for pancreatic cancer, Simpson et al.^36^ suggested that delta-radiomics texture features could be predictive of local control after the first fraction as well. Including this current study, GLCM energy was associated with treatment response in three different histologies^36,37^.

In this study, GLCM energy and GLSZM large zone emphasis were identified as predictive features for treatment response. Features identified earlier in treatment produced a stronger predictive signal than features later in treatment. These features increased in patients who responded to treatment between the beginning of treatment and delivery of 20 Gy BED (Fig. 3). Delivery of another 20 Gy BED resulted in a decrease in feature values between the 20 Gy BED and 40 Gy BED timepoints. Patients with no treatment response demonstrated opposite trends, with an initial decrease in feature values followed by an increase by the 40 Gy BED point. This suggests that a key event for SBRT cell killing occurs with the first treatment with diminishing returns. The premise for texture-based delta radiomics is based on the hypothesis that phenotype is a proxy for biologic behavior. GLCM energy appears to be an interesting feature identified across multiple histologies that could predict for SBRT response. We hypothesize this may be due to vascular endothelial damage and reactive acute inflammation, but correlative radiobiology studies will need to confirm the architectural changes.

There are several shortcomings to this exploratory work. Generalizability and reproducibility were limited by the number of patients enrolled and the heterogeneity of the cohort. Subgroup analysis based on histology was not possible due to lack of sufficient statistical power and will need to be clarified prior to making conclusions regarding clinical management. Given the proportion of patients with local control and local failure, approximately 2/3 with local control and 1/3 with local failure, the selection of 2/3 of patient data for each bootstrapped run is not guaranteed to represent the full patient library. While some iterations will result in over-fitted models, others will result in under-fitted models. For clarity, some models will be trained and tested on patients with local control while others are trained with local control patients and tested on patients with local failure. The number of bootstrapped iterations should balance out the smooth out the extreme cases. There is no guarantee though and an agnostic presentation of all iterations is therefore presented in Fig. 2. Only second order texture features were included in this analysis for calculation of delta-radiomics texture analysis because of interpretability and generalizability. Many higher order texture features include complicated image filtering and meaningful data can be obscured from investigators^38,39^. Additionally, second order texture features are included in most texture analysis studies with an established literature across many modalities and disease sites^40–44^. Finally, other texture features were excluded to increase the specificity of variables a priori, given the small cohort. Also, as in all radiomics texture analysis studies, results could be impacted by contouring variability. GTV contouring is a relatively subjective task and future studies need to develop a generalizable, regimented protocol for delineating GTVs on images or automated contouring algorithms could help mitigate variability. Generalizability and reproducibility were limited by the number of patients enrolled and the heterogeneity of the cohort. Baseline MR characteristics of different liver lesions are heterogeneous and present a challenge for texture analysis. For example, on non-enhanced T2 weighted imaging, liver metastases may appear as central regions of hypointense necroses surrounded by hyperintense rims of viable tumor. Whereas primary HCC may present as a heterogeneous, mixed signal lesion and intrahepatic cholangiocarcinomas (ICC) may appear as hyperintense lesions. Despite these heterogeneous presentations, delta radiomics may be able to discern a meaningful signal, and future studies will need clarification on this matter. Lastly, the binary nature of the response and use of RECIST 1.1 could smooth over the response data, and future analysis should aim to include measurable or observable biology for response determination, as well as actuarial outcomes.

## Conclusion

This preliminary MR-based delta-radiomics texture analysis study suggests that low field MR setup images may be able to capture meaningful phenotypic data, reflective of biologic changes seen as treatment response to SBRT. It may be possible, with expanded studies, to identify patients with liver malignancies treated with MRgSBRT at high risk for poor treatment response. The ability to identify non-responders prior to standard restaging scans three months after SBRT should not be understated and rigorously explored moving forward. Further work will be undertaken to include an expanded patient library and an external validation cohort, potentially leading to multi-institutional studies.

## Data Availability

The datasets generate during and/or analyzed during the current study are available from the corresponding author on reasonable request.
